# The utilization of chitosan/*Bletilla striata* hydrogels to elevate anti-adhesion, anti-inflammatory and pro-angiogenesis properties of polypropylene mesh in abdominal wall repair

**DOI:** 10.1093/rb/rbae044

**Published:** 2024-04-27

**Authors:** Yuntao Di, Lu Wang, Wei He, Shuyan Liu, Yuqi He, Jie Liao, Ruihong Zhang, Lan Yin, Zhiwei Xu, Xiaoming Li

**Affiliations:** Department of Neurosurgery, The Fourth Central Hospital of Baoding City, Baoding 072350, China; Research Center for Biomedical Engineering, Medical Innovation & Research Division, Chinese PLA General Hospital, Key Laboratory of Biomedical Engineering and Translational Medicine, Ministry of Industry and Information Technology, Beijing 100853, China; Key Laboratory for Biomechanics and Mechanobiology of Ministry of Education, Beijing Advanced Innovation Center for Biomedical Engineering, School of Biological Science and Medical Engineering, Beihang University, Beijing 100083, China; College of Lab Medicine, Hebei North University, Zhangjiakou 075000, China; Key Laboratory for Biomechanics and Mechanobiology of Ministry of Education, Beijing Advanced Innovation Center for Biomedical Engineering, School of Biological Science and Medical Engineering, Beihang University, Beijing 100083, China; Key Laboratory for Biomechanics and Mechanobiology of Ministry of Education, Beijing Advanced Innovation Center for Biomedical Engineering, School of Biological Science and Medical Engineering, Beihang University, Beijing 100083, China; Department of Neurosurgery, The Fourth Central Hospital of Baoding City, Baoding 072350, China; Key Laboratory of Advanced Materials of Ministry of Education, Tsinghua University, Beijing 100084, China; College of Lab Medicine, Hebei North University, Zhangjiakou 075000, China; Key Laboratory for Biomechanics and Mechanobiology of Ministry of Education, Beijing Advanced Innovation Center for Biomedical Engineering, School of Biological Science and Medical Engineering, Beihang University, Beijing 100083, China

**Keywords:** abdominal wall, wound healing, hydrogel, *Bletilla striata*, anti-adhesion

## Abstract

Polypropylene (PP) mesh is commonly used in abdominal wall repair due to its ability to reduce the risk of organ damage, infections and other complications. However, the PP mesh often leads to adhesion formation and does not promote functional tissue repair. In this study, we synthesized one kind of aldehyde *Bletilla striata* polysaccharide (BSPA) modified chitosan (CS) hydrogel based on Schiff base reaction. The hydrogel exhibited a porous network structure, a highly hydrophilic surface and good biocompatibility. We wrapped the PP mesh inside the hydrogel and evaluated the performance of the resulting composites in a bilateral 1 × 1.5 cm abdominal wall defect model in rats. The results of gross observation, histological staining and immunohistochemical staining demonstrated the positive impact of the CS hydrogel on anti-adhesion and wound healing effects. Notably, the addition of BSPA to the CS hydrogel further improved the performance of the composites *in vivo*, promoting wound healing by enhancing collagen deposition and capillary rearrangement. This study suggested that the BSPA-modified CS hydrogel significantly promoted the anti-adhesion, anti-inflammatory and pro-angiogenesis properties of PP meshes during the healing process. Overall, this work offers a novel approach to the design of abdominal wall repair patches.

## Graphical abstract

**Figure:**
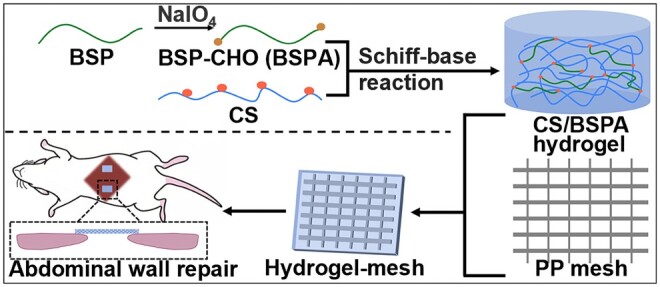

## Introduction 

The use of surgical meshes for abdominal wall repair has been a common practice to restore the integrity of the musculofascial layers. However, the rough surface of mesh patches could easily rub against visceral tissues, leading to adhesions [1–3] and resulting in poor healing or even failure of implantation due to complications such as visceral adhesions, pain from network contraction and bacterial infections [4–6], which still remains a tough clinical challenge [7, 8]. In general, wound healing is a complicated and dynamic process involving four main phases, which are hemostasis, inflammation, migration-proliferation and remodeling [9]. As a crucial role in wound healing, an ideal wound dressing is supposed to absorb tissue fluid, stop bleeding, get attached to the wound surface properly without sticking to the wound tissue, protect the wound from infection and promote wound healing. Up to now, researchers have explored various approaches to minimize the side effects of mesh repair and the drawbacks of the mesh have fueled interest in novel wound dressings such as hydrogels, films, nanofibers and sponges [10]. An effective practice is to wrap a layer of hydrogels outside the mesh [11–13]. Certain hydrogels have shown excellent performance in preventing cell adhesion while promoting tissue healing [14–17].

**Figure 1.**
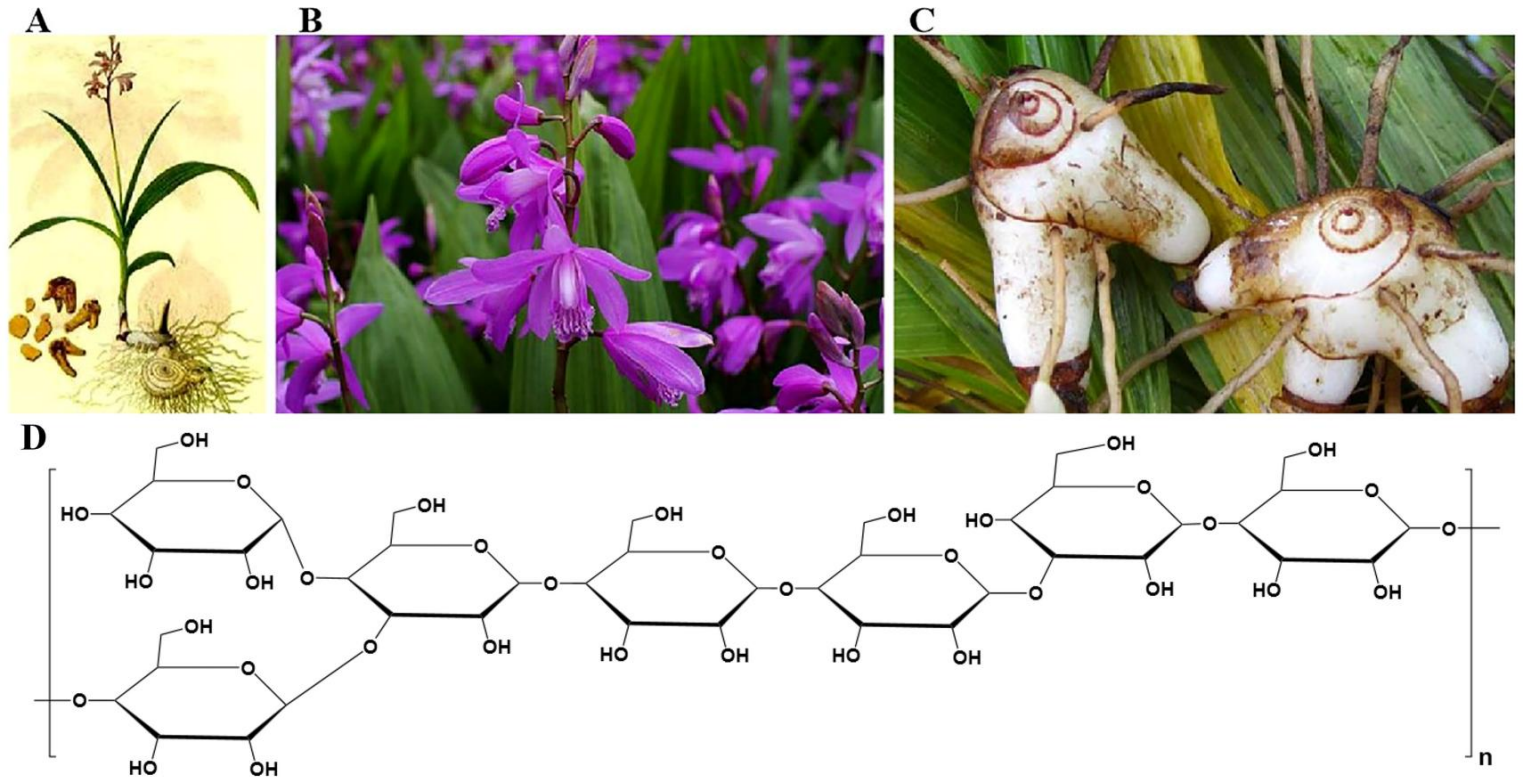
(**A**) *Bletilla striata* plant; (**B**) *Bletilla* flower; (**C**) *Bletilla* rhizome; (**D**) Molecular structure of *Bletilla* polysaccharide (Adapted from Refs. [24] and [25], with permission from Elsevier).

As one kind of natural polysaccharides, chitosan (CS) has shown superior performance in anti-adhesion and anti-bacterial activity [18, 19]. CS can be extracted and isolated from various seafood waste sources and has been extensively studied for its biomedical applications [20–23]. However, the lack of anti-oxidation properties in CS restricts its application in wound healing. Apart from that, the mechanical properties of pure CS film make it unsuitable for application in some biomedical settings. BSP, derived from the tuber of the traditional Chinese medicinal plant *Bletilla striata*, has been used extensively for clinical treatment [24] (Figure 1A–C), owing to its excellent antioxidative, anti-inflammatory and wound-healing properties [26, 27]. That is, the introduction of additional *B. striata* polysaccharide (BSP) may be effective in improving the performance of the pure CS film. Actually, BSP has shown prospects in wound dressing research [28–30], while its application in abdominal wall repair has received little attention. Nevertheless, it is difficult for physical mixture of CS and BSP to form a gel. Fortunately, BSP with multiple hydroxyl groups can be oxidized into aldehyde *B. striata* polysaccharide (BSPA), the aldehyde groups of which can react with the amino groups of CS (Figure 1D) through the Schiff base reaction to form an *in situ* cross-linked hydrogel. Other hydrogels formed by the reaction between aldehyde and amino groups have already been reported in the research of medical wound dressings [31, 32]. Therefore, the combination of CS and BSPA is expected to offer improved properties for mesh coating in terms of anti-adhesion and wound healing.

Herein, we synthesized a BSPA-modified CS hydrogel through the Schiff base reaction and utilized it to wrap a PP mesh and examined the microstructure, biocompatibility and hydrophilicity. To figure out the contribution of CS/BSPA hydrogel in the healing process, we implanted the hydrogel-mesh composites in the bilateral 1 × 1.5 cm abdominal wall defect in rats. And gross observation, histological staining and immunohistochemical staining were adopted to qualitatively and quantitatively characterize the effects of hydrogel and BSP on anti-adhesion, collagen deposition and angiogenesis, as well as the presence of inflammation.

## Materials and methods

### Materials

The CS (degree of deacetylation ≥ 95.0%), BSP and PP mesh were purchased from Shanghai Aladdin Biochemical Technology Co., Ltd, Xi'an Jincuifang Plant Technology Development Co., Ltd. and Shanghai Johnson & Johnson Medical Equipment Co., Ltd, respectively.

Mouse embryonic fibroblasts (NIH-3T3) were purchased from Beijing Dingguo Biotechnology Co., Ltd.

Other reagents used in the study, including chemicals, solvents, buffers and other materials necessary for *in vitro* and *in vivo* experiments, were purchased from domestic manufacturers.

### Preparation of BSPA

BSPA was synthesized through an oxidation reaction using sodium periodate. Following the reaction, the product underwent precipitation, centrifugation and drying processes. Subsequently, the titration methods employing hydroxylamine hydrochloride solution and sodium hydroxide solution were utilized to determine the aldehyde group content in BSPA. Fourier transform infrared spectroscopy (FTIR) was adopted to confirm the occurrence of oxidation reaction. Thermogravimetry (TG) was analyzed to detect the thermal stability.

### Fabrication and characterization of the CS/BSPA hydrogel

**Figure 2.**
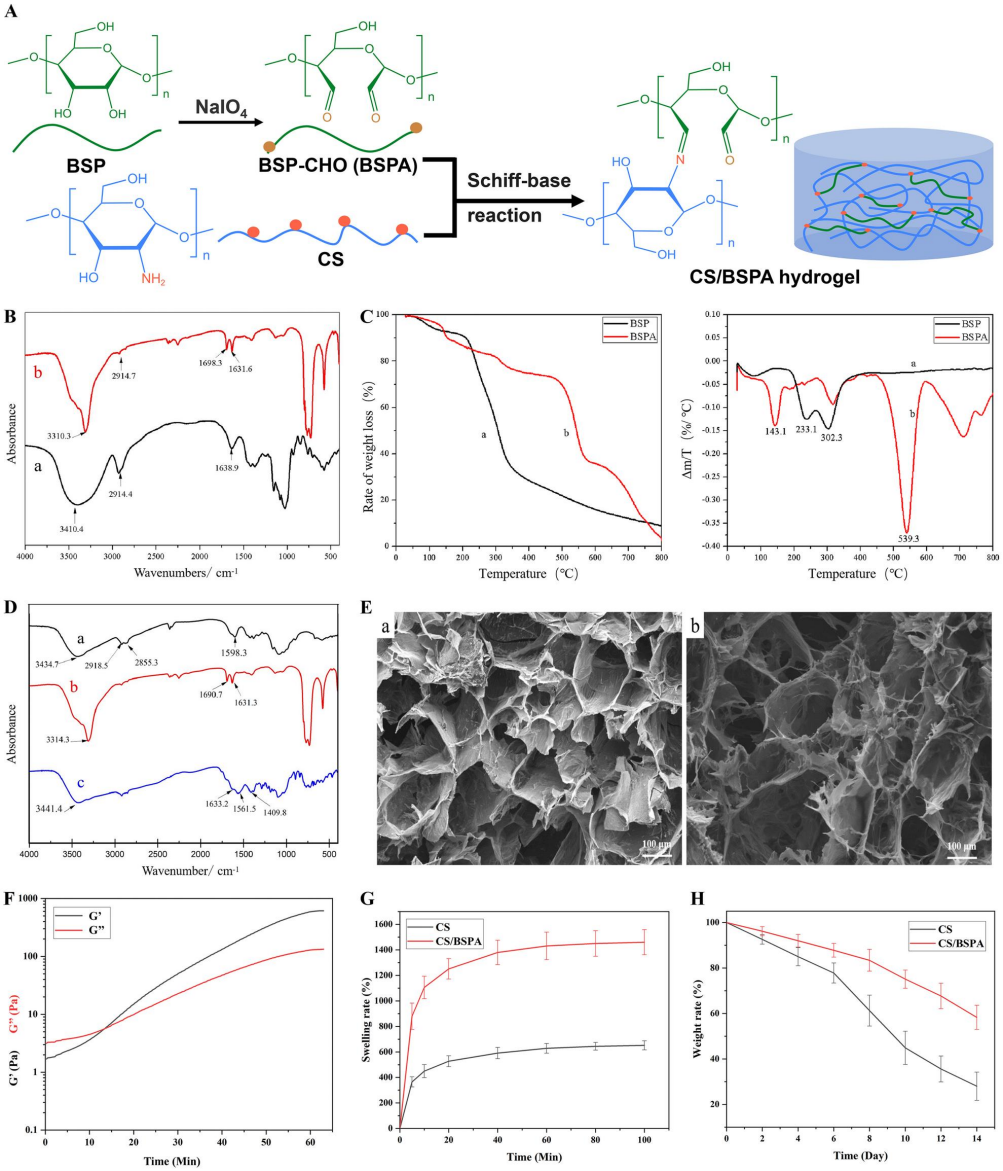
(**A**) Preparation and reaction process of the CS/BSPA hydrogel; **(B**) FTIR curves of BSP (a) and BSPA (b); (**C**) TG (left) and DTG (right) curves of BSP and BSPA; (**D**) FTIR curves of CS (a), BSPA (b) and CS/BSPA (c); (**E**) SEM images of the hydrogels (a: CS, b: CS/BSPA); (**F**) Rheological results of the CS/BSPA hydrogel; (**G**) The swelling rate of CS/BSPA and CS hydrogels; (**H**) The weight rate of hydrogels during the degradation *in vitro*.

To prepare the CS/BSPA hydrogel, CS solutions with different weight percentages (2 wt% and 4 wt%) were mixed with BSP or BSPA solutions (10 wt%) in varying proportions (1:1, 2:1 and 3:1), as depicted in Figure 2A. This mixing process was carried out drop by drop using a syringe, while stirring conditions were maintained. The sol-gel process was then observed, and the gelling time was obtained by rheological analysis (MCR 302, Anton Paar). The storage modulus (G’) and loss modulus (G″) of the CS/BSPA hydrogel were measured (frequency = 1 Hz).

To prepare the CS hydrogel as a control, we adjusted the pH of the chitosan solution to the range of 5.5–5.8 with acetic acid solution. Then, the saturated sodium carbonate solution was added into the obtained CS solution until the pH rose to the range of 6.5–7.0. After being stirred for a while, the CS solution was allowed to stand until the gel process was finished.

The hydrogel's pore micromorphology was characterized using a Scanning Electron Microscopy (SEM). The presence of Schiff base formation was detected using FTIR. Furthermore, the hydrophilicity of the hydrogels was examined by measuring the contact angle. We also investigated the *in vitro* degradation and swelling behavior (in a PBS solution, pH 7.0–7.4, 37°C) of the hydrogels that would be used in subsequent *in vivo* experiments. The detailed methods could be seen in previous literatures [30, 32].

### *In vitro* cell culture experiment

According to the ISO 10993-5:2009 standard, we used the extraction method to conduct the *in vitro* cytotoxicity experiment of all groups. The extraction conditions were designed to simulate the *in vivo* environment. Concretely, to prepare the extracts, we added the complete culture medium to the sterilized hydrogels or PP mesh in a centrifuge tube. The mixing ratio was 0.1 g/ml. The mixture was then gently shaken at 37°C for 24 hours to allow for proper diffusion of the components. The complete culture medium of the blank control group was treated through the same extraction conditions described above. Afterwards, the extracts were collected and filtered using a sterile filter membrane with a pore diameter of 0.22 μM.

Next, NIH-3T3 cells were seeded into a 48-well plate. The extract from each group was added, with three replicates per group (*n* = 3). The cells were then cultured for 1 day, 3 days and 5 days, respectively, at 37°C and 5% CO_2_. To evaluate the cytotoxicity of the hydrogel, we performed a CCK-8 assay. The viability and morphology of the cells were assessed using DAPI staining and Phalloidin-TRITC staining. To observe cell growth on both the surface and inside of the hydrogel, we added 300 μL of the cell suspension to a 48-well plate after formation and sterilization of hydrogels and incubated the cells for 1 day and 3 days, respectively, at 37°C and 5% CO_2_. And DAPI staining was performed to visualize the cells.

HUVECs (Human Umbilical Vein Endothelial Cells) tube formation assay was conducted to explore the angiogenesis potential of the hydrogels. In brief, HUVECs were seeded onto the sample of each group in 96-well plates pre-coated with Matrigel (Corning, USA). The cells were incubated at 37°C for 6 h. And then, tube formation was observed using a fluorescence microscope (LSCM, LSM800, Zeiss, Germany).

### Preparation of hydrogel-mesh composite

The PP mesh was cut into small pieces measuring 15 mm × 20 mm, and each piece of the PP mesh was placed inside a 6 cm diameter petri dish. The prepared CS solution or the mixed solution (containing CS and BSP/BSPA) was slowly poured into the petri dish, ensuring that the solution spread out evenly around the PP mesh patch. Then, the composite was kept at room temperature for a specific duration. After the designated time, the hydrogel-mesh composite was prepared, which had a thickness of ∼2–3 mm.

### Biological evaluation of CS/BSPA hydrogel-PP composites in vivo

Twenty-four male SD rats aged 8–10 weeks and weighing 200–250 g each were obtained from Beijing Weitong Lihua Laboratory Animal Technology Co., Ltd. All the animal procedures were conducted under the Guidelines for Care and Use of Laboratory Animals of Beihang University and ratified by the Animal Ethics Committee of Biology and Medicine at Beihang University (BM201900117).

The experiment consisted of the following groups: the CS/BSPA hydrogel-mesh group, the CS hydrogel-mesh group, the PP mesh and the non-surgical group. There were nine cases in each of the CS/BSPA and CS hydrogel-PP composite groups. Each rat had the hydrogel-mesh implanted on one side of the abdominal wall and the PP mesh patch as a control on the other side. Additionally, three rats were implanted with hydrogel-mesh composite patches as backup samples. Three rats of the non-surgical group were prepared for comparison purposes. Preoperative preparations and procedures for the implantation surgery could be briefly described as follows:

The rats were anesthetized using an intraperitoneal injection of a 0.3% pentobarbital solution in the proportion of 40–45 mg/kg. Then, bilateral full-thickness abdominal wall defects, measuring 1 cm × 1.5 cm, were created on both the left and right sides of the abdomen. The defects were covered with either a hydrogel-mesh composite or a pure PP mesh patch, depending on the experimental groups being studied. Afterwards, the patch was sutured securely to the nearby abdominal muscles and the abdominal skin was closed with sutures. After the designated time intervals of 1, 2 and 4 weeks, the rats were euthanized to observe the adhesion between the abdominal wall and intestinal tract, as well as wound healing of the defects. The collected samples were placed into a 4% histopathological fixation solution to preserve their cellular and tissue structures, followed by being fixed using the paraffin embedding method. Histological and immunohistochemical staining were performed to observe the angiogenesis, collagen deposition and inflammation. Moreover, we used the Image J software for quantitative analysis.

### Statistical analysis

The data in this study were presented as mean ± SD (standard deviation). To determine if there were any statistical differences between the groups, one-way ANOVA and student's t-test were used. A statistically significant difference was defined as a *P* values <0.05.

## Results and discussion

### Characterization of physicochemical property

#### Characterization of aldehyde group

Figure 2B showed the spectrum of BSP. It was observed that the BSP curve exhibited a wide absorption peak at 4310.4 cm^−1^. This peak was attributed to the stretching vibration of the hydroxyl group in the polysaccharide. In the BSPA curve, an additional absorption peak was present at 1698.3 cm^−1^. This peak represented the stretching vibration of the aldehyde group, indicating that an oxidation reaction had occurred. The BSPA in this study had an aldehyde group content of ∼13.7 mmol/g, which was determined using hydroxylamine hydrochloride solution titration. These aldehyde groups were able to react with the amino groups in the CS structure through a Schiff base reaction, leading to the formation of an *in situ* cross-linked hydrogel. This sol-gel mechanism has been applied in previous studies [25, 29, 31].

#### TG analysis

In Figure 2C, the TG curve for BSPA was more complex compared to BSP, displaying a stepwise weight loss pattern. Between 20°C and 110°C, the TG curve showed a gradual decrease with a mass loss of ∼5%. This weight loss was primarily attributed to the removal of water molecules bound to polysaccharides and residual impurities resulting from oxidation reactions. In the range of 260–650°C, the TG curve demonstrated a two-step reduction mode. The weight loss rate during this stage was ∼50%. The first peak, accounting for a 10% weight loss, was attributed to the breakage and thermal decomposition of the main chain. The second peak occurred at 539°C and indicated an accelerated thermal degradation. This acceleration could be caused by the presence of aldehyde functional groups, which delayed the thermal degradation of the main chain, resulting in a weight loss rate of 40%. Overall, the results indicated that BSPA exhibited a higher weight loss rate, more complete thermal decomposition and better initial thermal stability compared to BSP.

#### Sol-Gel time and microstructure

The sol-gel experiment revealed that untreated BSP was unable to spontaneously form the hydrogel when mixed with CS. Thus, we observed the sol-gel behavior based on different concentrations of CS and the addition ratio of BSPA.

The results of the mixing experiment indicated that when 2 wt% CS and 10 wt% BSPA were combined in volume ratios of 1:1, 2:1 and 3:1, the gelling process did not occur effectively even after ∼24 h at room temperature. The resultant mixture did not acquire a fixed form and instead exhibited a mucus-like shape. This outcome suggested that the chosen combination of 2 wt% CS and 10 wt% BSPA, in the specified volume ratios, did not lead to the desired hydrogel formation.

After increasing the concentration of CS solution to 4 wt% and mixing it with 10 wt% BSPA in the same volume ratio, the sol-gel time decreased to 6 hours, 2 hours and 1 hour for volume ratios of 1:1, 2:1 and 3:1, respectively. Among these groups, we found that the hydrogels formed from the 1:1 and 2:1 volume ratios still had flowability, suggesting that they were not fully solidified. Meanwhile, the hydrogel formed from the 3:1 volume ratio was solid and could be separated from the mold, indicating a complete gelation process. And, it was calculated that the molar ratio between the aldehyde and amino group of this experimental group was ∼1:1. As reflected in the rheological results in Figure 2F, the gelling process started after 15 minutes or so and finished in ∼60 min, which was consistent with the above experimental observations. Based on these data, the CS/BSPA group with a volume ratio of 3:1 was selected for further experiments *in vitro* and *in vivo*. This group demonstrated promising gelation properties and structural stability, making it suitable for subsequent investigations.

After the lyophilization process, SEM was performed, and Figure 2E showed the resulting images. The SEM images revealed the presence of micropores inside the samples, ranging in size from 100 to 300 μm. The presence of these interconnected reticular pores in the hydrogel was advantageous for both tissue migration and water retention. Overall, the addition of BSPA did not significantly alter the pore characteristics of the hydrogel. Furthermore, the CS/BSPA group exhibited a higher number of smaller pores compared to the CS group, which could be attributed to the formation of a more complex inner network as a result of the Schiff base reaction between CS and BSPA. These smaller pores may play a vital role in the repair of abdominal wall tissue involving bioactive substances [33].

#### Schiff-base reaction

The introduction of aldehyde groups onto the polysaccharide chain of BSP through oxidation enabled the formation of a hydrogel through a Schiff base reaction with the amino group of CS. One significant advantage of this gel preparation method was that it avoided the use of toxic crosslinking reagents such as glutaraldehyde and formaldehyde.

The Schiff base reaction could be analyzed using FTIR, as shown in Figure 2D. In the top black curve, characteristic peaks could be observed. A small peak at 3434.7 cm^−1^ corresponded to the stretching vibration of -NH and -OH groups, indicating the presence of CS. Peaks at 2925 cm^−1^ and 2856 cm^−1^ represent the symmetric and antisymmetric stretching vibrations of—CH_2_ groups [32]. On the red curve, a prominent wide absorption peak was visible at 4310.4 cm^−1^, which corresponded to the stretching vibration of the -OH group in polysaccharides. Another smaller absorption peak at 2914.4 cm^−1^ was caused by the stretching vibration of C-H bonds. The absorption peak at 1638.9 cm^−1^ indicated the asymmetric stretching vibration of the acetyl group. Additionally, a wide and strong absorption band between 1000 and 1200 cm^−1^ represented the stretching vibration of the C-O-C glycosidic bond and the C-O-H terminal group. In the FTIR spectral curve of the CS-BSPA hydrogel (blue line at the bottom), several characteristic peaks were observed. First, there was a vibration peak of hydroxyl groups at 3434.1 cm^−1^, similar to that observed in the other curves. Additionally, two peaks were visible at 1561.5 cm^−1^ and 1409.8 cm^−1^, corresponding to the stretching vibration of amide NH (amide II) and HN-CO (amide III) groups [34]. These peaks indicated the presence of amide bonds in the hydrogel structure. Compared to the other two curves, a new peak appeared at 1633.2 cm^−1^ in the CS-BSPA hydrogel curve. This peak corresponded to the vibration of C = N in the imine bond, confirming the occurrence of the Schiff base reaction [35, 36]. This indicated that the aldehyde group on BSPA had reacted with the amino group of CS, forming the Schiff base linkage. Furthermore, the absorption peak at 1690.7 cm^−1^, which corresponds to the aldehyde group in BSPA, was absent in the composite hydrogel curve. This disappearance of the peak further confirmed the occurrence of the CS/BSPA Schiff-base reaction. These spectral observations provided evidence for the successful formation of the Schiff base linkage between CS and BSPA, resulting in the formation of the CS-BSPA hydrogel.

#### Swelling behavior and degradation in vitro

With the addition of BSPA, the swelling rate increased to ∼1400% after the sample was soaked in the PBS for one hour, while the swelling rate of CS hydrogel was ∼610%, as shown in Figure 2G. The swelling behavior of the hydrogel in this study was consistent with the data in previous research literature [30]. The different swelling behavior was due to the cross-linking structure present in BSPA hydrogel, which would affect the pore structure and porosity of the gel and in turn change the swelling properties of the hydrogel. The swelling behavior would have certain positive effect on the *in vivo* performance of this implant. For example, the obvious swelling performance might indicate a better absorptive capacity of excessive tissue fluid at the wound site and be beneficial to the healing process.

Meanwhile, the degradation of the BSPA hydrogel was significantly slowed down, due to the presence of cross-linking structures, as exhibited in Figure 2H. After one week, the weight loss of the CS group was ∼30%, about twice that of the CS/BSPA group. In two weeks, more than half of the CS/BSPA hydrogel group remained, while the CS group remained only about one-quarter of the initial weight. The low weight loss value of hydrogel during the degradation *in vitro* indicated the ability of the hydrogel to maintain structural integrity and stability after implantation [34]. In other words, the degradation rate of the CS/BSPA hydrogel could better adapt to the needs of tissue reconstruction process *in vivo* than that of the CS hydrogel.

### *In vitro* cytocompatibility and angiogenesis

**Figure 3.**
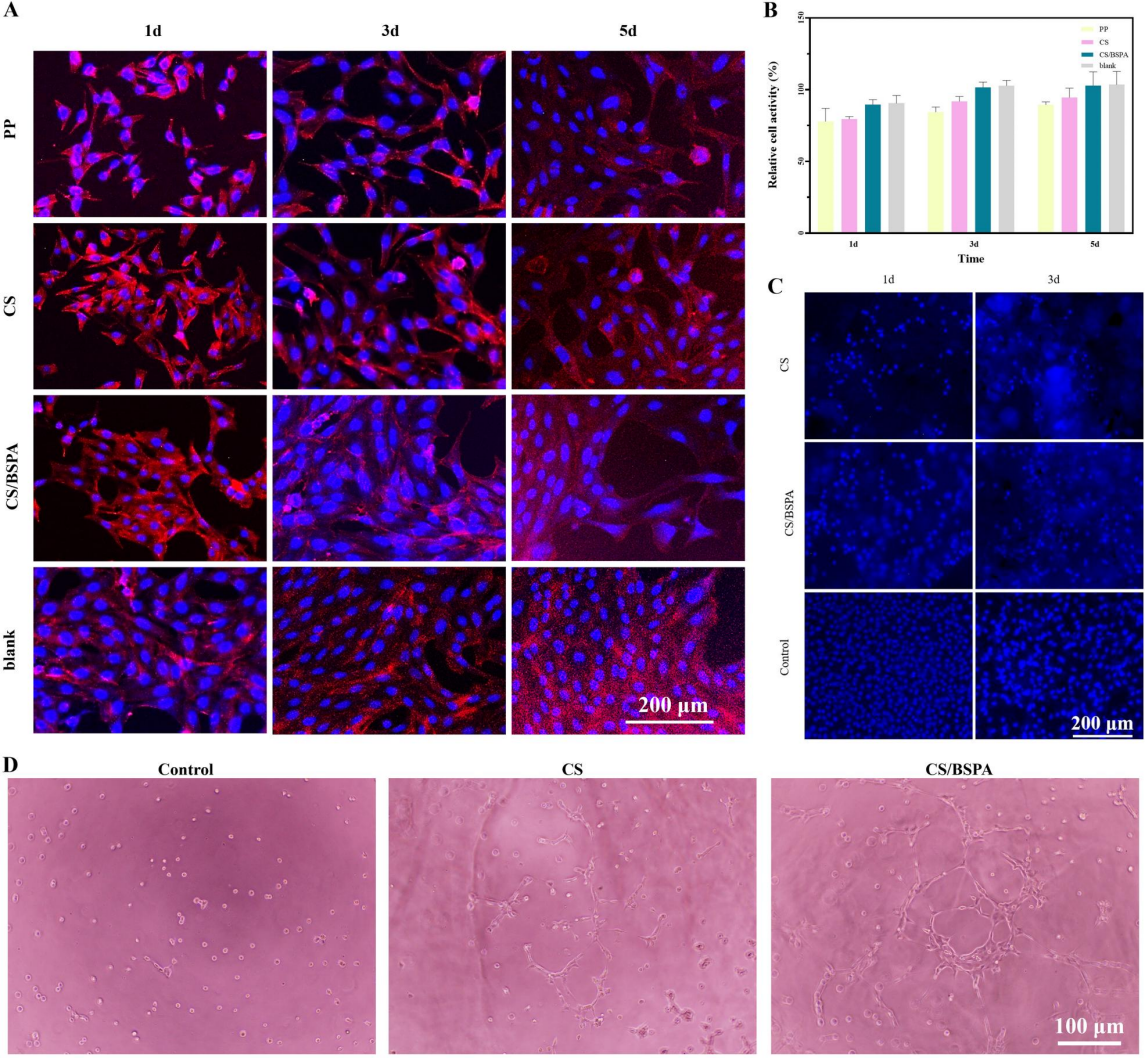
(**A**) Fluorescence plots after DAPI and Phalloidin-TRITC staining of NIH-3T3 cultured in extracts for 1, 3 and 5 days; (**B**) Relative cell activity after cultured in extracts of the hydrogel and PP mesh groups (*n* = 3); (**C**) Fluorescence plots of cells stained with DAPI after cultured on the hydrogel and control groups; (**D**) HUVECs tube formation on the hydrogel and control groups.

The biocompatibility of biomaterials is a crucial factor to consider before their implantation into human tissues or organs [37]. According to the observations depicted in Figure 3A, spindle-shaped cells in all groups of extracts exhibited robust proliferation and adherence to the pore plates. The cells displayed a long spindle shape with well-defined cytoskeleton structures. Over the third and fifth days, the cytoskeleton gradually extended, becoming more evident, and a significant number of pseudopods were observed. The cell distribution in the PP mesh control group appeared relatively sparse compared to the other groups, while the cells in the remaining groups exhibited a more compact arrangement. This suggested that the extract from the mesh control group had some negative impact on cell growth, although it remained within a safe range, as determined by the cytotoxicity test. Overall, these results indicated that the hydrogel extracts possessed appropriate cellular compatibility.

The relative activity of cells cultured in extracts of the hydrogel and PP group for 1 day, 3 days and 5 days was presented in Figure 3B. The cell activity in each group exceeded 75% (between 75% and 90%), with the CS/BSPA group approaching 100% after 3 days of culture. According to ISO 10993-5:2009, the cytotoxicity grade of all groups was assessed as Grade I, which indicated that the materials could be safely used within the organism. Furthermore, there were no significant differences observed between the results at 3 and 5 days, suggesting that the materials exhibit stable biocompatibility over a certain period. These results implied that all groups met the safety requirements for cell activity.

DAPI staining was performed to observe cell growth on the surface and inside of the hydrogel. In Figure 3C, cell growth in the hydrogel groups and the control (complete medium) group was shown. On the first day, there were relatively fewer cells observed in the hydrogel groups compared to the blank group where the cells filled the bottom of the pore plate, which might be because the cells needed more time to adapt to the hydrogel material by establishing constant and adequate contact. On the third day, there was no significant difference in the number of cells between the CS group and the CS/BSPA group. However, different levels of fluorescence intensity were observed, indicating that the cells had started to migrate into the gel. The cells in the CS/BSPA group appeared denser, and both hydrogel groups showed evidence of cell migration into the gel. These findings suggested that the hydrogel materials, both in the CS group and the CS/BSPA group, support cell growth and migration. The DAPI staining provided insights into the distribution and behavior of cells within the gel, demonstrating their ability to penetrate and populate the hydrogel matrix.

Angiogenesis is a vital process for normal tissue development and wound healing. To explore the angiogenesis potential of CS and BSPA, we conducted HUVECs tube formation experiments (Figure 3D). Endothelial cells proliferate through *in situ* differentiation to form a preliminary vascular network structure. And the tube formation is an important indicator to analyze the pro-angiogenic ability of materials [38]. As expected, all the hydrogel groups showed obvious effects on tube formation and the CS/BSPA group appeared as the more effective one, which was probably attributed to the enhanced migration and proliferation of endothelial cells induced by BSP.

### Appearance and microstructure of hydrogel-mesh composites

**Figure 4.**
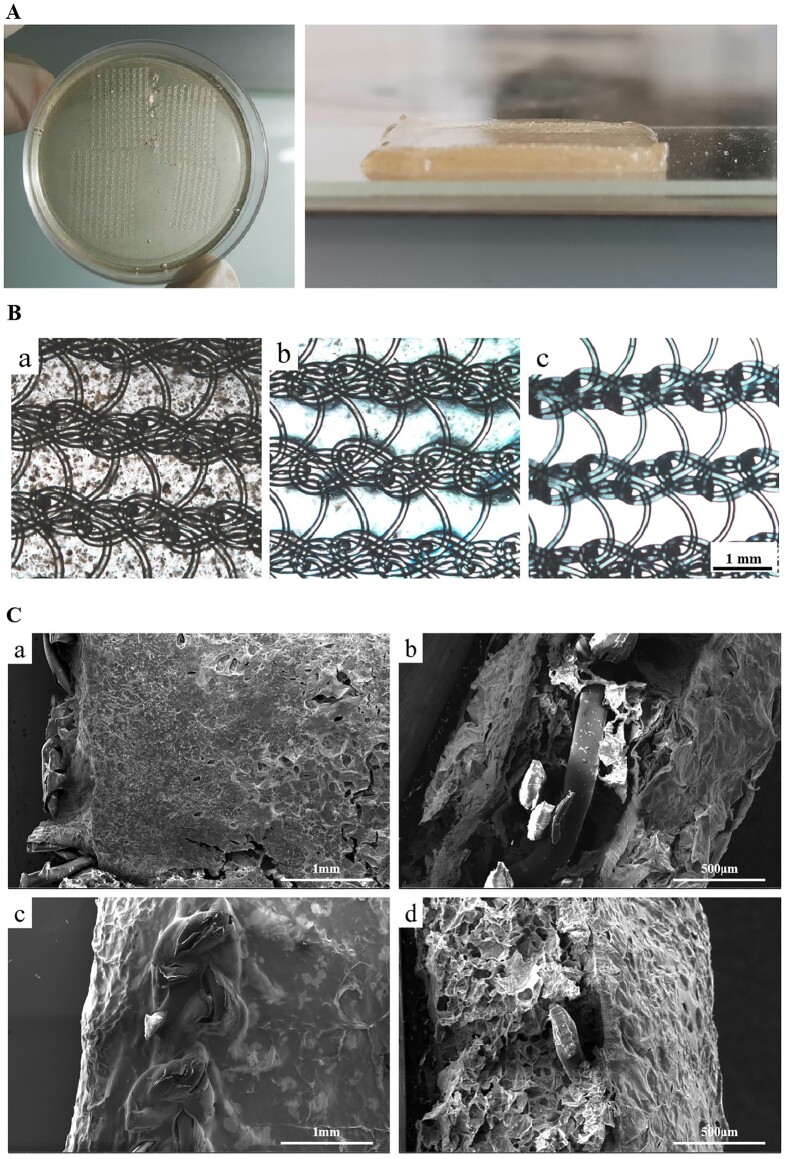
(**A**) Appearance of the hydrogel-mesh composites; (**B**) Micrographs of the hydrogel-mesh composites (a: CS/BSPA-PP, b: CS-PP) and the pure PP mesh (c); (**C**) SEM images of the hydrogel-mesh composites [CS-PP (a: front view, b: side view); CS/BSPA-PP (c: front view, d: side view)].

As depicted in Figure 4A, the hydrogel membrane demonstrated a light-yellow transparent appearance. The PP mesh was wrapped inside the hydrogel and could not be easily peeled off. This wrapping effect was intended to effectively prevent the shedding of the hydrogel and facilitate the suturing of the hydrogel at the defects during subsequent implantation. Under the optical microscope, as shown in Figure 4B, it was evident that the hydrogel was tightly wrapped by the polypropylene mesh, forming a closely woven network structure. This observation confirmed the successful integration of the hydrogel with the mesh material. Furthermore, within the CS/BSPA group, the internal particles were evenly distributed without obvious impurities or bubbles. This uniform distribution ensured the quality and integrity of the composite material, indicating that the synthesis process was well-controlled and that the resulting hydrogel-mesh composite exhibited a consistent and homogenous structure. Overall, these figures demonstrated a promising wrapping effect of the hydrogel around the PP mesh, providing potential benefits for preventing hydrogel shedding and facilitating subsequent suture implantation [39]. Additionally, the even distribution of particles within the CS/BSPA group confirmed the high quality and purity of the composite material.

To gain a clearer understanding of the internal microstructure, freeze-drying treatments were conducted on the hydrogel-mesh composites before SEM observation. The results, as depicted in Figure 4C, further confirmed the firm wrapping of the PP mesh within the hydrogels. In Figure 4C(a), the protruding edge of the PP mesh could be observed, which was a result of the cutting process during sample preparation. However, it was evident that the main parts of the PP mesh remained completely wrapped by the hydrogel. Figure 4C(c) shows some cracks in the hydrogel caused by shrinkage during the freeze-drying process. However, these cracks were not expected to affect the actual *in vivo* use due to the intact and strong structure of the PP mesh. The cutting profile of the hydrogel after lyophilization, as seen in Figure 4C(b, d), confirmed that the hydrogel had penetrated the mesh of the PP mesh, forming a porous structure. This observation was consistent with the presence of reticular pores in the hydrogel shown in Figure 2E. Importantly, there was no significant change observed in the reticular pores of the hydrogel, indicating that the porous properties of the hydrogel itself were not significantly affected before and after the PP mesh was wrapped. Overall, these results showed that the PP mesh was securely wrapped within the hydrogel, forming a composite material with a porous structure. The intact and strong structure of the PP mesh ensured the stability and effectiveness of the composite, while the porous properties of the hydrogel remained unchanged.

### Evaluation of the hydrogel-mesh composite in repairing abdominal wall defects

#### The ability to resist adhesion

**Figure 5.**
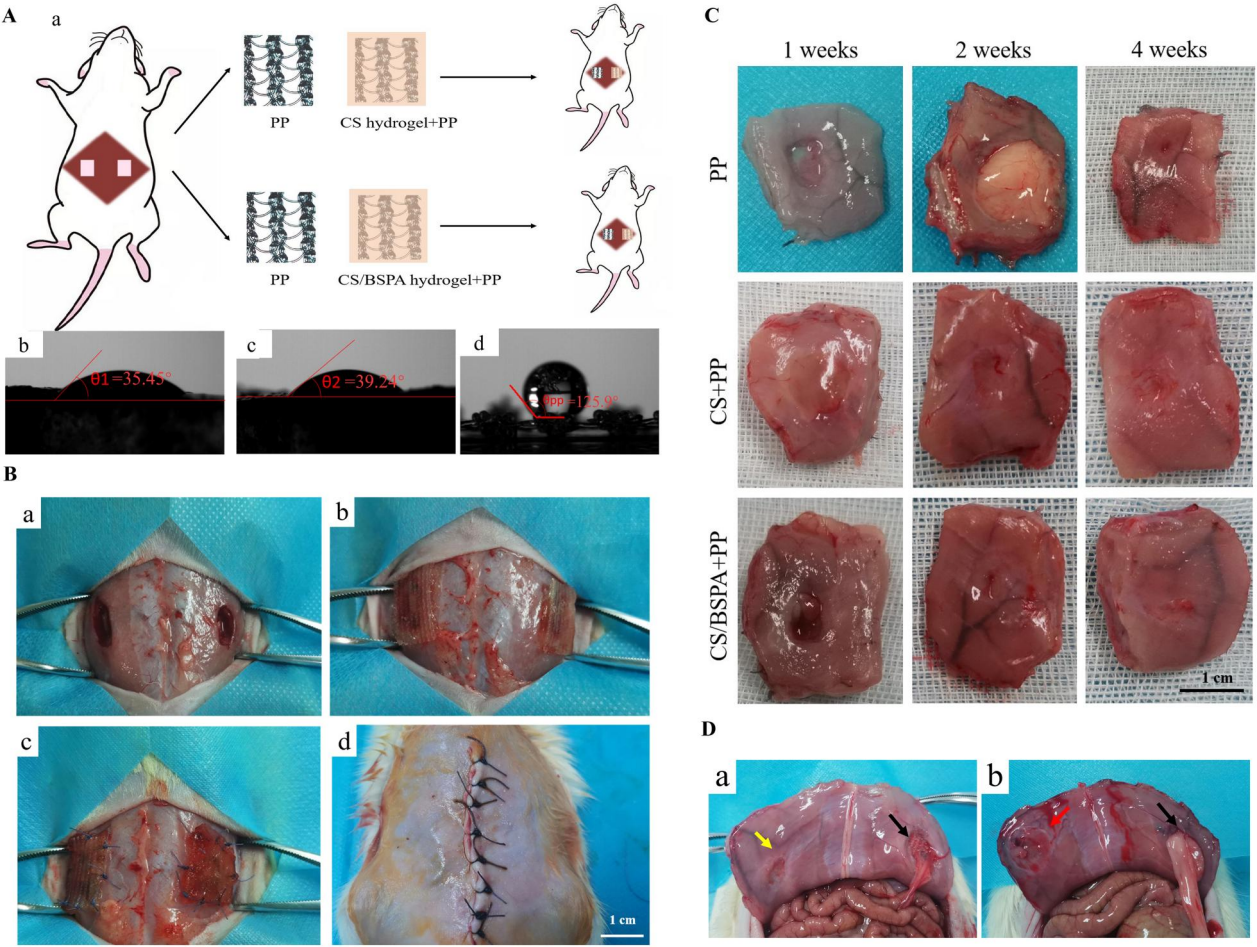
(**A**) Schematic representation of implantation in defects (a) and hydrophilicity reflected by the contact angle measurement [(b) CS/BSPA group; (c) CS group; (d) PP mesh group]; (**B**) Surgical procedures for the repair of the abdominal wall defects; (**C**) General observation of the defects after implantation of different patches; (**D**) General observation inside abdominal wall after 2 weeks of implantation [the right arrow indicates the pure PP mesh group; (a) the left arrow indicates the CS/BSPA-PP composite group; (b) the left arrow indicates the CS-PP composite group].

In Figure 5A, the contact angle measurement of the PP mesh resulted in a value of 125.9 ± 0.4°, significantly >90°. This indicated that the surface of the PP mesh was highly hydrophobic. Water droplets added to the surface of the PP mesh would persist for a long time, suggesting that the PP mesh was not conducive to the absorption of blood and tissue fluid when implanted in the body. Conversely, the contact angle of the CS/BSPA group was measured to be 39.24 ± 0.3°, and the contact angle of the CS group was measured to be 35.45 ± 0.6°. These values indicated that the hydrogels possessed significant hydrophilicity. When water was dropped onto the surface of the hydrogel, it spread and disappeared within a few seconds, indicating strong water absorption and hydrophilicity of the hydrogel. The outer layer of hydrogel wrapping the mesh served to enhance its hydrophilicity. Based on these results, it was expected that when the hydrogel-mesh composites were implanted into the body, they would effectively prevent adhesion. The hydrophilic nature of the hydrogel, combined with the hydrophobicity of the PP mesh, could create an anti-adhesive effect. Further verification of this hypothesis was conducted *in vivo*.

After one week of implantation, the pure PP mesh group showed no tissue ingrowth, with only partial coverage by the mucosa. In contrast, in the hydrogel group, tissue and blood vessels started to grow, indicating initial tissue regeneration. The CS-PP group exhibited some connective tissue covering the defect, which might be attributed to the incomplete absorption of early tissue fluid. In the CS/BSPA-PP group, a small amount of tissue grew inside the composite and the defect area contracted, suggesting good wound healing. After two weeks, as depicted in Figure 5C and D, the PP mesh in the control group adhered to the abdominal fat tissue and could not be separated easily. The presence of adipose tissue obstructed the abdominal gap, which hindered further healing of the abdominal tissue. The integrity of the abdominal wall muscle tissue could not be restored. In contrast, in the hydrogel-mesh groups, the tissue at the defects showed evident healing without any adhesion. Numerous new tissues and microvessels were observed at the defects. After four weeks, the wounds in the control group (PP mesh) exhibited obvious healing; however, there were still noticeable gaps in the defect, indicating that the defect was not fully healed. The integrity of the abdominal wall was not restored. Conversely, in the hydrogel-mesh group, the original traces of the defect were almost invisible, and there was no tissue adhesion. Abundant new blood vessels and tissues were present, and the abdominal wall muscles were reconstructed, restoring the anatomical morphology of the abdominal wall. These results highlighted the superior healing properties and tissue regeneration capabilities of the hydrogel-mesh group compared to the control group with only the PP mesh. The hydrogel promoted proper tissue growth, prevented adhesion and enabled the restoration of the abdominal wall structure.

Indeed, individual differences among rats could result in varying outcomes in the PP mesh control group, with some experiencing adhesion and others not. If adhesions occurred between the mesh and the fat layer within one or two weeks, normal tissue regeneration was hindered by the presence of adhesions at the fourth week, leading to persistent adhesion. Conversely, if no adhesions occurred in the initial stages, it was expected that tissue regeneration would continue without impediment in the later stages. Prior studies have demonstrated that PP mesh could stimulate surrounding tissues after implantation, which might induce the adhesion of organs and tissues [40]. The hydrogel, however, could alleviate this stimulation, thereby reducing the likelihood of adhesion and promoting the normal regeneration of abdominal muscle tissue. As depicted in Figure 5C, the hydrogel wrap not only protected internal organs but also facilitated wound healing. Particularly, after two or four weeks, the degree of wound healing in the hydrogel groups surpassed that observed in the pure PP mesh group. These results affirmed the beneficial effects of the hydrogel in preventing adhesion and supporting the healing of abdominal wounds. By reducing the stimulation caused by the PP mesh, the hydrogel promoted better wound-healing outcomes.

#### The ability to promote wound healing in defects

##### Histology and immunohistochemistry analysis

The hydrogel for wound dressing was usually expected to sustain a proper microenvironment for the healing process and promote re-epithelialization and granulation tissue formation [41]. Thus, in addition to the performance of anti-adhesion, we further examined the hydrogel’s capability of facilitating cell-infiltration, collagen deposition and angiogenesis after implantation, which is vital during the wound-healing procedure.

**Figure 6.**
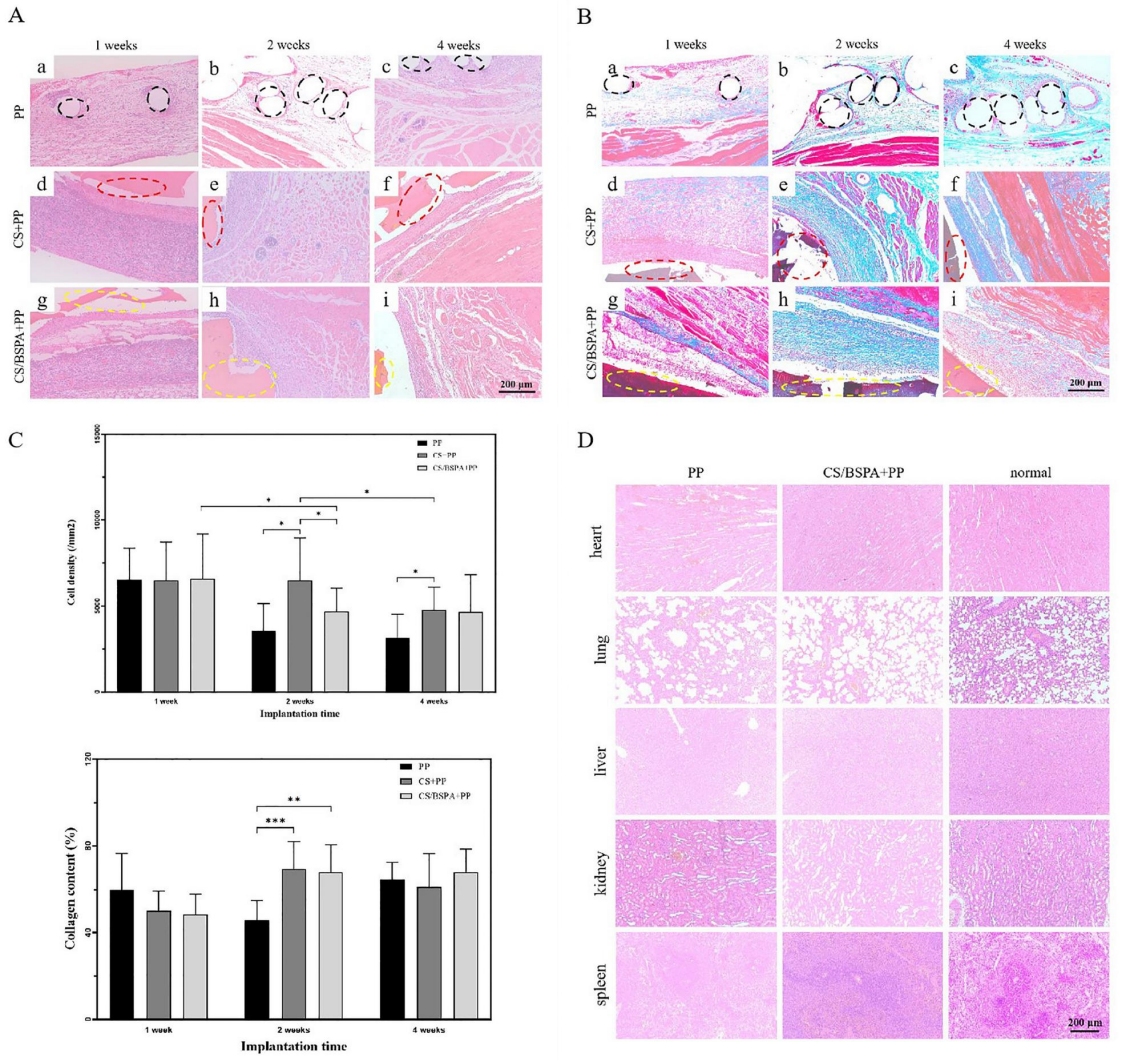
(**A**) Representative histological images stained with HE at different time points after implantation; (**B**) Representative histological images stained with Masson, highlighting collagen deposition and tissue remodeling (the dashed lines in (a-c) demarcated the area of the PP mesh, while the dashed lines in (d-i) indicated the presence of undegraded hydrogels); (**C**) Analysis of cell density at defects after implantation in different groups (above), quantitative analysis of collagen content after implantation in different groups (below) (*n* = 3); (**D**) Histological images illustrating the condition of intracorporal organs at the fourth week after implantation.

In this study, HE staining was used to investigate cellular infiltration. As shown in Figure 6A, there remained undegraded hydrogel at the fourth week, indicating that the hydrogels could adapt to the need for stability during the tissue repair process. After one week, a significant amount of cell infiltration was observed in all groups, along with a noticeable inflammatory response and capsule encapsulation. After two or four weeks, the fibrous capsule was still present, while cell infiltration decreased. Angiogenesis and new abdominal wall muscle cells were visible. The hydrogel coating exhibited a promoting effect on tissue regeneration and remained stable even after long-term implantation. After two and four weeks, the inflammatory response decreased due to wound generation, blood vessel injury and foreign body stimulation caused by the implantation. Cells were primarily located inside the PP mesh, with fewer surrounding cells. In contrast, cell infiltration in the hydrogel-mesh composites was weaker and mainly observed between the hydrogel and muscle tissue. As depicted in Figure 6C (above), the cell density in all groups was ∼6500 cells/mm^2^ after one week of implantation, showing no significant difference. After two weeks, the cell density decreased in the pure mesh group, while there was no change in the CS-PP group. The CS/BSPA-PP group exhibited a significant decrease in cell density. After four weeks, all groups showed a decrease in cell density to varying degrees. These results indicated that the PP mesh group had the lowest level of cell infiltration, while the hydrogel group exhibited higher levels of cell infiltration. This suggested that the implantation of the hydrogel stimulated the recruitment of cells at the implantation site, and this recruitment effect continued over time, which promoted the subsequent regeneration of related tissues.

To further investigate the biological mechanism of the repair process, the deposition of collagen in the granulation tissue was evaluated based on the results shown in Figure 6B and C (below). Type I collagen, muscle and cytoplasm were stained blue, red and pink, respectively. Following the implantation, all implants were surrounded by a layer of fibrillar collagen-like tissue. After one week, significant fibrosis occurred around the implantation site in all groups, leading to the production of a large amount of collagen fibers. At two weeks, the area occupied by collagen fibers in the hydrogel-mesh composite groups was larger than that in the pure PP mesh group, indicating that the implantation of the hydrogel induced and promoted the deposition of more collagen. In addition, a large number of living fibroblasts were observed in the wound site, suggesting that the hydrogels could supply an appropriate matrix and microenvironment for the growth, proliferation and migration of fibroblasts [42]. The collagens were mainly distributed in and around those fibroblasts, which indicated the fabrication capability of the collagen from the fibroblasts. By the fourth week, the content of collagen at the implantation site was similar among all groups. Re-deposition of collagen was observed in the PP mesh group. Notably, the content of collagen in the CS/BSPA-PP group was higher than that in the CS-PP group. These results suggested that the implantation of the hydrogel promoted collagen deposition, with a greater effect observed compared to the pure PP mesh group. Furthermore, the CS/BSPA-PP group exhibited superior collagen deposition compared to the CS-PP group. In addition to matrix deposition, the directional alignment of collagen fibers was also essential for improving extracellular matrix construction.

In terms of collagen distribution, as shown in Figure 6B, after one week, the collagen distribution in each group appeared random and disorganized, without clear orientation. After two weeks in the PP mesh group, loose connective tissue appeared evident. In contrast, the hydrogel-mesh composite groups exhibited collagen fibers arranged in an orderly manner with better orientation, probably owing to the contribution of CS in regulating the repair process of wound healing [43]. These observations illustrated that the hydrogel-mesh composites could promote collagen deposition, alignment and organization. The presence of BSP in the composite would positively facilitate the wound-healing process.

The metabolites of the material *in vivo* and the potential impact on the main organs are of great importance in evaluating the safety of implants for clinical applications. After four weeks, the visceral organs (heart, lung, liver, kidney and spleen) of the PP mesh group and CS/BSPA-PP group were examined by using HE staining, as depicted in Figure 6D. No macroscopic lesions or abnormalities were observed in any of the organs. There was no significant difference between the different groups and the normal rats, indicating that the implanted materials did not exhibit significant toxicity to the visceral organs. These findings suggested that the PP mesh, hydrogels and the concomitant degradation products were biocompatible and did not cause any apparent harm or adverse effects to the visceral organs at the fourth week after implantation.

##### Immunohistochemistry analysis

**Figure 7.**
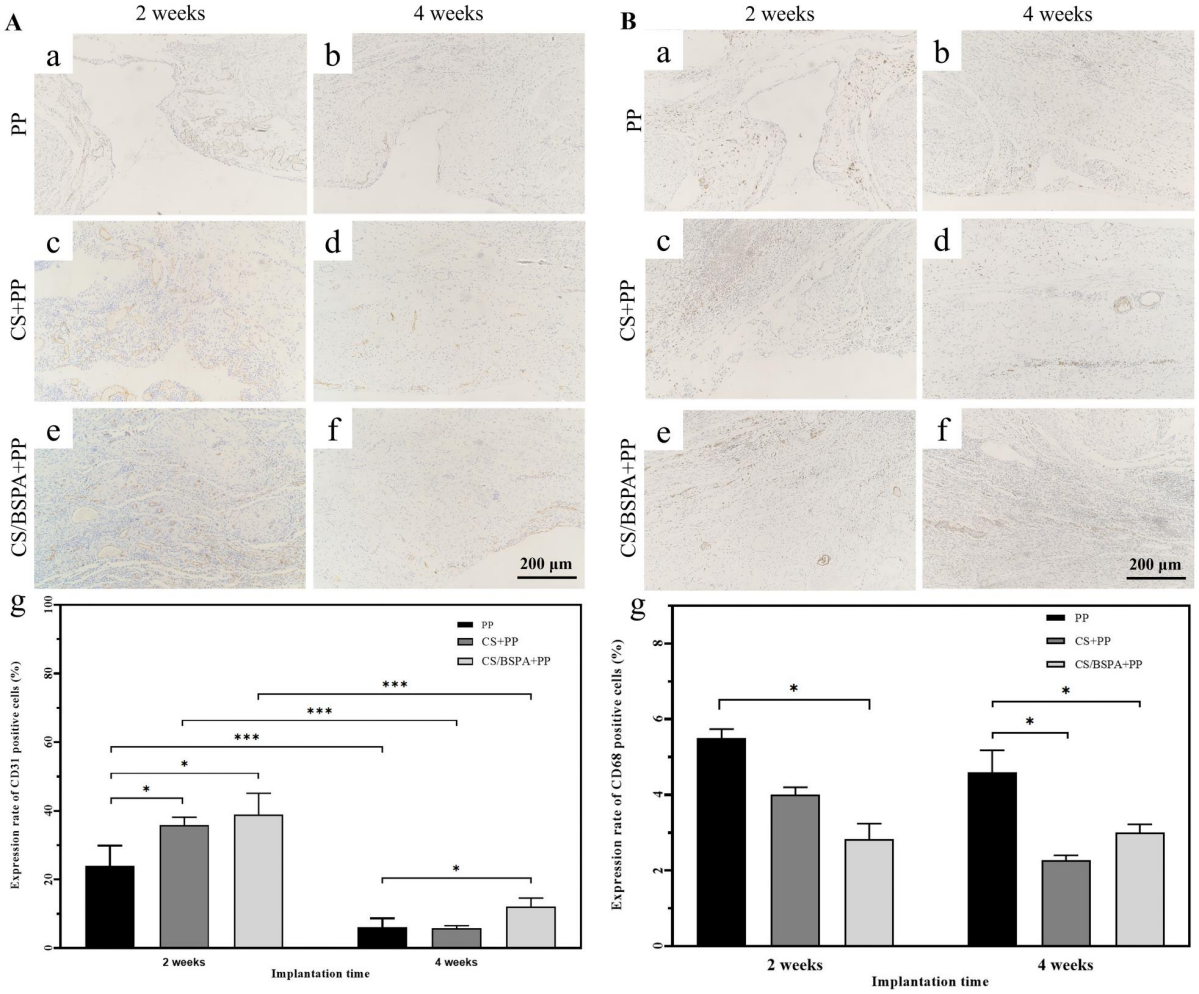
(**A**) Illustration of immunohistochemical staining showing the presence of CD31, a marker for angiogenesis. The expression ratio of CD31-positive cells is quantified (*n* = 3); (**B**) Illustration of immunohistochemical staining displaying the presence of CD68, a marker for macrophage infiltration. The expression ratio of CD68-positive cells is quantified (*n* = 3).

From the analysis of CD31 staining images, it could be found that the blood vessel density in the wound bed was significantly increased in the CS/BSPA-PP group. As shown in Figure 7A, after two weeks, CD31-positive cells were concentrated around the defects, indicating the presence of numerous new blood vessels involved in tissue reconstruction. Notably, in the PP mesh group, large defects could be observed, with a significant concentration of new capillaries at the site. In contrast, in the hydrogel-composite group, cells within the abdominal wall defect had already completed the process of proliferation and reconstruction. Capillaries were distributed between the new muscle layers, supplying nutrients and oxygen to facilitate subsequent reconstruction. By the fourth week, CD31-positive cells were no longer centrally distributed. Instead, positive cells could be observed between the muscle layers and the inner surface of the abdominal wall. This suggested that the reconstruction process of the abdominal wall had been completed essentially, and the various cellular tissues tended to be structurally stable. Capillaries were primarily concentrated on the surface of the inner membrane of the abdominal wall. Interestingly, the expression rate of CD31-positive cells appeared higher in the CS/BSPA-PP group compared to other groups. This suggested that BSP might stimulate the formation of blood vessels during the repair process. In contrast, the pure PP mesh group still exhibited obvious gaps, which differed from the results observed in the hydrogel-mesh composite groups. Immunohistochemical staining of CD31 revealed potential angiogenesis effect of the hydrogel-mesh composite, which could contribute to the reconstruction of the abdominal wall. And the presence of BSP in the composite would further enhanced blood vessel formation.

Besides, macrophages also play an important role in tissue repair and remodeling during the wound repair process. Thus, CD68 immunohistochemical staining was performed to detect macrophage-related inflammatory reactions induced by the implants. As shown in Figure 7B, at two weeks after implantation, a significant number of inflammatory cells were observed in all groups. CD68-positive cells were densely clustered in the abdominal wall defect and the contact area between the mesh and the site. This could be attributed to the wound-healing process initiated by the body after the abdominal wall injury. Macrophages quickly emerged to clean up apoptotic cells and impurities at the wound site. Monocytes gradually infiltrated the wound bed since the onset of inflammation and transformed into activated macrophages, making them crucial cells in the wound healing process [44–46]. Macrophages also played a role in recruiting vascular endothelial cells to prepare for angiogenesis. There have been reports suggesting that macrophages could also attract fibroblasts and smooth muscle cells to the wound site, promoting collagen matrix deposition and wound tissue healing [47–50]. After four weeks, CD68-positive cells were concentrated between the intima of the abdominal wall and the newly formed muscle tissue layer, indicating ongoing inflammatory reaction and tissue reconstruction. Between the two hydrogel-mesh groups, the number of CD68-positive cells decreased more rapidly in the CS-PP group, indicating relief of inflammation at the wound healing site and active tissue healing. In contrast, the number of CD68-positive cells in the CS/BSPA-PP group remained at a lower level, with no significant difference observed between two weeks and four weeks. These findings suggested that the inflammatory reaction persisted and tissue reconstruction was still underway after four weeks. The CS-PP group exhibited faster resolution of inflammation, indicating active tissue healing. The CS/BSPA-PP group maintained a lower level of CD68-positive cells, suggesting a relatively more controlled and regulated inflammatory response throughout the healing process.

## Conclusions

In this study, a hydrogel, composed of CS and a traditional Chinese medicine named BSP, was synthesized based on Schiff base reaction to optimize the anti-adhesion and wound healing performance of the PP mesh. And the hydrogel-mesh composites were prepared to investigate the potential contribution to improving the performance of pure meshes in abdominal wall repair. The results demonstrated that the hydrogels exhibited good biocompatibility and hydrophilicity, with favorable porous micro-structural conditions for cellular growth inside. Through *in vitro* and *in vivo* biological experiments, hydrogel-mesh composites’ abilities to resist adhesion and promote wound healing were verified. Fundamentally, the experimental results in this study demonstrated that the hydrogels contributed positively to the anti-adhesion and wound-healing effects of the composites. Specifically, the BSP addition could further enhance the anti-adhesion, anti-inflammatory and pro-angiogenesis properties during the healing process. Generally, this research have provided new insights into the introduction of hydrogels with traditional Chinese medicine BSP in abdominal wall repair, offering promising possibilities for addressing the challenges associated with abdominal adhesion and reconstruction.
